# Dietary Soy Consumption and Cardiovascular Mortality among Chinese People with Type 2 Diabetes

**DOI:** 10.3390/nu13082513

**Published:** 2021-07-23

**Authors:** Xiaowen Wang, Jun Lv, Canqing Yu, Liming Li, Yonghua Hu, Li-Qiang Qin, Jia-Yi Dong

**Affiliations:** 1 Department of Epidemiology and Biostatistics, School of Public Health, Peking University Health Science Center, Beijing 100191, China; wangxw@bjmu.edu.cn (X.W.); lvjun@bjmu.edu.cn (J.L.); yucanqing@bjmu.edu.cn (C.Y.); lmlee@vip.163.com (L.L.); yhhu@bjmu.edu.cn (Y.H.); 2 Public Health, Department of Social Medicine, Osaka University Graduate School of Medicine, Osaka 5650871, Japan; 3 Department of Nutrition and Food Hygiene, School of Public Health, Soochow University, Suzhou 215000, China; qinliqiang@suda.edu.cn

**Keywords:** soy, prospective study, diabetes, cardiovascular disease, mortality

## Abstract

Randomized controlled trials showed that soy intervention significantly improved blood lipids in people with diabetes. We sought to prospectively examine the association of soy consumption with the risk of cardiovascular death among individuals with diabetes. A total of 26,139 participants with a history of diabetes were selected from the Chinese Kadoorie Biobank study. Soy food consumption was assessed by a food frequency questionnaire. Causes of death were coded by the 10th International Classification of Diseases. The Cox proportional hazard regression was used to compute the hazard ratios. During a median follow-up of 7.8 years, a total of 1626 deaths from cardiovascular disease (CVD) were recorded. Compared with individuals who never consumed soy foods, the multivariable-adjusted risks (95% confidence intervals) of CVD mortality were 0.92 (0.78, 1.09), 0.89 (0.75, 1.05), and 0.77 (0.62, 0.96) for those who consumed soy foods monthly, 1–3 days/week, and ≥4 days/week, respectively. For cause-specific cardiovascular mortality, significant inverse associations were observed for coronary heart disease and acute myocardial infarction. Higher soy food consumption was associated with a lower risk of cardiovascular death, especially death from coronary heart disease and acute myocardial infarction, in Chinese adults with diabetes.

## 1. Introduction

Soy is rich in plant protein, soy isoflavones, vitamins, dietary fiber, and minerals [1]. Numerous randomized controlled trials (RCTs) have examined the effects of soy intervention on cardiovascular risk factors. Meta-analyses of trials showed that soy or soy isoflavones significantly improved blood pressure [2], blood lipids [3], and endothelial function [4]. Furthermore, there is evidence from observational studies showing an inverse association with cardiovascular disease (CVD) risk when comparing the highest versus lowest intake levels of soy [5,6].

Clinical trials have shown that soy intervention significantly improved blood lipids in people with type 2 diabetes, although blood glucose, insulin, and glycated hemoglobin were not affected [7]. The lipid-lowering effects of soy consumption in people with type 2 diabetes suggested a potential role in CVD prevention in this population [8]. However, evidence regarding the association between soy and CVD incidence or mortality in people with type 2 diabetes is sparse, with only one Singapore study on this topic [9]. To fill this knowledge gap, in the current analysis, we aimed to examine whether soy consumption is associated with CVD mortality among individuals with type 2 diabetes in the Chinese Kadoorie Biobank (CKB) cohort.

## 2. Methods

### 2.1. Study Population

**Figure 1:**
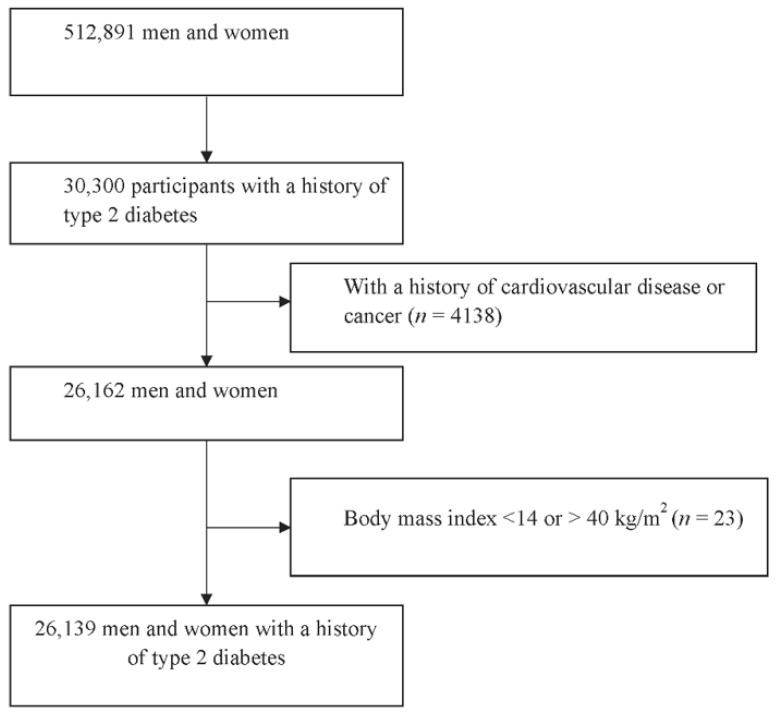
Flow chart of participant selection.

The population with a history of type 2 diabetes was selected from the CKB study. The CKB study was a large nationwide prospective cohort study aiming to identify risk factors of non-communicable diseases and provide evidence for prevention in the Chinese population. The detailed study design has been reported previously [10,11]. Briefly, 512,891 individuals (after data quality control) aged 30–79 years were recruited from 2004–2008 at baseline, from 5 urban and 5 rural areas in China. For this investigation, a total of 30,300 participants who reported a history of type 2 diabetes (16,413 participants) or had random blood glucose ≥ 11.1 mmol/L or had a fasting blood glucose ≥ 7.0 mmol/L (13,887 participants) were defined as having a history of type 2 diabetes. We excluded 4138 people who reported a history of cancer, stroke, transit ischemic attack, or coronary heart disease (CHD). We further excluded 23 participants having a body mass index (BMI) < 14 or > 40 kg/m^2^. Finally, a total of 26,139 individuals were eligible in the current analysis (Figure 1).

The Ethics Review Committee of the Chinese Center for Disease Control and Prevention (CDC), the Oxford Tropical Research Ethics Committee, the University of Oxford, and the institutional research boards at the local CDCs approved the study. All participants have provided written informed consent.

### 2.2. Assessment of Diet

Diet was assessed by a food frequency questionnaire (FFQ) with 12 food groups including soy products, rice, wheat, other staple foods, meat, poultry, fish/seafood, fresh eggs, vegetables, preserved vegetables, fruits, and dairy products. Participants were asked how often they ate soy products as well as other foods during the past 12 months. Soy products included soybean, tofu, fried tofu, pressed tofu, soy milk skins, soy milk, and other kinds of soy products. Five categories were provided: daily, 4–6 days/week, 1–3 days/week, monthly, and never/rarely. We combined the highest two categories as regular consumers (≥4 days/week) because of a limited number of participants. To evaluate the reproducibility and validity of soy product consumption in the CKB study, 432 participants were selected to complete two FFQs (median interval: 3.3 months) and twelve 24 h dietary recalls in 2015–2016, which covered all kinds of soy foods in China. The weighted kappa coefficient was 0.66 for reproducibility and 0.67 for validity of baseline soy product consumption [12].

### 2.3. Assessment of Covariates

Each participant enrolled in the CKB study was asked about socio-demographic characteristics (sex, age at recruitment, education level, marital status, occupation, household income), personal and family history of major diseases (cancer, CVD, diabetes, hypertension, etc.), lifestyle (smoking status, drinking status, tea consumption, dietary habits, physical activity), sleeping, and mental health using a standard questionnaire. Height and weight were obtained from physical examination by trained staff. BMI was then calculated as (weight in kg)/(height in meters)^2^.

### 2.4. Follow-Up

Official residential records and death certificates were used to monitor the vital status of each participant [13]. Causes of death were coded by the 10th International Classification of Diseases. Death from any CVD (I00–I99) was the main outcome of this analysis. Cause-specific CVD mortality, including death from CHD (I20–I25), acute myocardial infarction (I21), stroke (I60–I69), ischemic stroke (I63), and hemorrhagic stroke (I61), were also examined. Pulmonary heart disease, unspecified stroke type, and other vascular disease were grouped as other CVDs.

### 2.5. Statistical Analysis

Baseline characteristics of participants with a history of type 2 diabetes were computed according to frequencies of soy product consumption. Person-years of follow-up were computed from the date of participation until the date of loss to follow-up, death, or 31 December 2014, whichever came first. The Cox proportional hazard regression was used to compute the hazard ratios (HRs) and 95% confidence intervals (CIs) of CVD mortality associated with soy product consumption. The HRs were adjusted for age first and then, in a multivariable model, were adjusted for sex, study area (urban or rural), BMI (quintile), family history of CVD (yes or no), history of hypertension (yes or no), drinking status (never or almost never, occasionally, at certain seasons, every month, at least once a week), smoking status (non-smoking, occasionally, most days, daily), education level (no formal school, primary school, middle school, high school, college, university), household income (6 categories), occupation (10 categories), marital status (married, divorced, widowed, or never married), physical activity (quintile), vitamin supplement use (yes or no), fish oil use (yes or no), consumptions of dairy foods, tea, eggs, fresh fish, fresh fruits, fresh vegetables, meat, and rice (5 categories). The *p* value for linear trends was assessed by considering the soy consumption as a continuous variable. Stratification analyses according to age, sex, BMI, smoking status, drinking status, region, and hypertension status were performed to test whether these factors could modify the association between soy product consumption and cardiovascular mortality, which was tested by adding to the model a cross product term between covariates and soy consumption. Sensitivity analyses with further adjustment for age at diabetes diagnosis, excluding participants who died within the first 3 years of follow-up, and adding antidiabetic and lipid-lowering therapy in the multivariate models were conducted. All analyses were carried with the use of SAS software (version 9.4). A *p* value < 0.05 was considered statistically significant.

### 2.6. Patient and Public Involvement

Patients were not involved in the study design, conduct, setting, reporting, or dissemination plans of our research.

## 3. Results

Of the 26,139 participants, 15,999 (61.2%) were women. Baseline characteristics of participants with type 2 diabetes according to soy product consumption are presented in Table 1. Regular consumers were less likely to be women and farmers, but more likely to be current smokers and current drinkers, live in urban areas, have a higher education level and household income, family history of CVD, and history of hypertension, use fish oil and vitamin supplements, and have higher consumptions of dairy, eggs, fish, fruit, vegetables, meat, and rice than participants who never consumed soy.

During a mean follow-up of 7.8 years, we documented 1626 deaths due to total CVD, including 595 due to CHD (305 acute myocardial infarction), 743 due to stroke (383 hemorrhagic and 227 ischemic stroke), and 288 due to other CVD. The frequency of soy consumption was inversely associated with CVD mortality among adults with type 2 diabetes in both the age- and multivariable-adjusted models. Compared with individuals who never consumed soy foods, the multivariable-adjusted risks (95% CIs) of CVD mortality were 0.92 (0.78, 1.09), 0.89 (0.75, 1.05), and 0.77 (0.62, 0.96) for those who consumed soy monthly, 1–3 days/week, and ≥4 days/week, respectively. Among cause-specific CVD mortality, this inverse association with soy consumption was evident for CHD (HR = 0.56 (0.39, 0.81) for regular consumers) and acute myocardial infarction (HR = 0.44 (0.26, 0.73) for regular consumers). Soy consumption was not associated with risk of death from stroke and stroke subtypes (Table 2).

We then conducted stratified analyses by age, sex, BMI, smoking status, drinking status, region, and hypertension status. Overall, age, sex, smoking, drinking, region, and hypertension status did not significantly modify the association between soy product consumption and cardiovascular mortality among participants with type 2 diabetes (all *p* for interaction > 0.10); BMI, however, appeared to be a potential modifier for the association (Table 3). The difference in multivariable-adjusted risks among those with a BMI < 25 kg/m^2^ and those with a BMI ≥ 25 kg/m^2^ reached statistical significance (0.57 (0.43, 0.76) vs. 1.12 (0.80, 1.56), *p* for interaction = 0.03).

In a sensitivity analysis, we further adjusted for years of diabetes history (*n* = 13,181), but the observed associations were not significantly changed. For example, the risk of CVD mortality for regular consumers was 0.77 (0.59, 1.01). In another sensitivity analysis excluding participants who died within the first 3 years of follow-up (*n* = 25,219), the risk of CVD mortality for regular consumers was 0.78 (0.60, 0.99). In addition, further adjusted for antidiabetic and lipid-lowering therapy changed the results little, for example, the risk of CVD mortality for regular consumers was 0.78 (0.63, 0.97) (Supplementary Table S1).

## 4. Discussion

The present cohort study provided evidence that higher consumption of soy products was inversely associated with risk of mortality due to CVD, especially CHD and acute myocardial infarction, in people with type 2 diabetes. BMI was a potential modifier as a significant lower risk of CVD mortality was observed among participants with a BMI < 25 kg/m^2^ but not among those with a BMI ≥ 25 kg/m^2^.

Soy consumption has been shown to be beneficial for many diseases, including cancers, diabetes, and hypertension [14]. Our study provided the first evidence that higher soy food consumption was associated with a lower risk of CVD death in people with type 2 diabetes. However, previous cohort studies examining the association between soy or soy isoflavone consumption and risk of CVD in general populations have produced controversial results. In line with our findings, an early cohort study of 40,462 Japanese people reported that compared with 0–2 times/week consumers, the risks (95% CIs) of cerebral infarction risk and CVD mortality were 0.64 (0.43 to 0.95) and 0.31 (0.13 to 0.74), respectively, for soy intake at least 5 times/week in women [5]. However, subsequent cohort studies conducted in Spain, Singapore, and Japan did not find this association between total soy product or soy isoflavone consumption and CVD mortality [9,15,16,17]. The discrepancy across studies may be attributable to different population characteristics, soy intake levels or measurement, and cardiovascular endpoints. For example, measurement error in soy intake might occur because there are various cooking methods and combinations with other foods for soy products, especially in Chinese cuisine, probably leading to different nutrient effects on health.

The mechanisms by which soy consumption was associated with a lower risk of CVD mortality among people with diabetes were uncertain. Soy protein intervention, compared with placebo, significantly reduced low-density lipoprotein cholesterol (LDL-C), total cholesterol, and total triglyceride in patients with type 2 diabetes in individual RCTs [18,19,20]. A meta-analysis of these trials conducted in people with pre-diabetes or type 2 diabetes confirmed a lipid-lowering effect of soy protein intervention [21], which was in line with the findings of a previous meta-analysis [7] and another meta-analysis conducted in the general population [3]. It has been well established that dyslipidemia is a major contributor to the increased CHD risk in patients with type 2 diabetes [22], while high blood pressure is the strongest risk factor for stroke among Asians [23]. Therefore, the lipid-lowering effect of soy intervention may be responsible for the inverse association between soy consumption and atherosclerotic CVD, especially CHD mortality, in the population with diabetes in our study. Unfortunately, we have no data on lipid profiles and were therefore not able to test whether the associations were mediated by lipid profiles. In addition, patients with diabetes might have discrepant salivary amylase content [24] or gut microbiomes [25] that probably have an effect on soy metabolism. Therefore, the observed association might be different within the general population.

We observed that the association of soy consumption with cardiovascular mortality was more evident in participants with a BMI < 25 kg/m^2^. This was consistent with the findings of the Shanghai Women’s Health Study in China, where soy consumption was associated with a decreased risk of CHD only in women with a BMI < 25 kg/m^2^ (HR = 0.16 (0.05–0.54)) but not in those with a BMI ≥ 25 kg/m^2^ (HR = 0.59 (0.18–1.91)) [26]. The underlying mechanisms for the observed difference remained unclear. A previous meta-analysis of 46 trials showed that soy protein intervention produced a somewhat greater reduction in circulating LDL-C as well as total cholesterol concentration among participants with a baseline level of LDL-C < 135 mg/dL [27]. Therefore, non-obese individuals, who were more likely to have a low level of LDL-C, may be more sensitive to the lipid-lowering effect of soy protein, which might decelerate the deterioration of CVD. Another possible explanation might be that obese individuals tended to over-report their healthy dietary intake, including soy foods, contributing to the null association among this population [28]. In addition, the interaction between the subgroup with BMI < 23 kg/m^2^ and BMI ≥ 23 kg/m^2^ (cut-off for Asian populations) did not reach significance, and chance remained an alternative explanation for this finding.

The main strengths of our study included a large sample size and a prospective cohort design. Limitations of the present study should also be noted. Firstly, our findings may have been affected by confounding bias. In the baseline survey, soy consumption was associated with a higher socioeconomic status in terms of educational achievement, household income, and occupation. Additionally, participants with high soy consumption were more likely to live in urban areas, and region heterogeneity may contribute to differences in environmental factors, lifestyle, and health care infrastructure. It was, therefore, possible that people having higher soy consumption may be associated with better treatment and medical care after diabetes diagnosis [29], which could affect the risk of CVD mortality. Nevertheless, our stratification analyses indicated that region did not significantly modify the association. Secondly, the FFQ in the CKB study only covered 12 major food groups instead of individual food items. Hence, it was not comprehensive enough to estimate energy intake or macronutrient composition, and it also precluded analyses for specific soy foods as well as soy isoflavones and application of substitution modeling and dose–response analysis. The bias of measurement errors also occurred due to a single baseline measurement only and due to the relatively broad exposure categories by using the FFQ. Thirdly, people at higher risk of death may have changed their dietary habits (may have increased their soy consumption at baseline survey). Yet, we excluded participants who died within the first 3 years of follow-up and obtained similar results. Fourth, assessments of type 2 diabetes at baseline were not entirely based on biological examination. However, since the age of most of the participants ranged from 40–79 years, the misclassification of other types of diabetes was minimal. Furthermore, the accuracy rate was found to be 98.6% after reviewing the medical records of a random sample of 831 self-reported diabetes cases [30,31]. Finally, whether our findings could be generalized to other populations, particularly the Western population with lower soy consumption, was uncertain given the differences in genetic background, lifestyle, and medical systems between China and other countries. Future studies could also explore the all-cause mortality in patients with diabetes though CVD is the most prevalent cause of mortality in people with diabetes. Nevertheless, the potential health effects of soy consumption provided support for dietary recommendations for the prevention of CVD death among Chinese people with type 2 diabetes, which has been a huge public health burden [32].

In conclusion, this protective cohort study provided evidence that higher soy food consumption was associated with a lower risk of cardiovascular death, especially CHD death, in Chinese adults with type 2 diabetes.

## Figures and Tables

**Table 1:** Baseline characteristics of individuals with a history of type 2 diabetes according to soy consumption *.

	Never	Monthly	1–3 days/week	≥4 days/week	*p* Trend
No. of participants	2416	6518	13,811	3394	
Age, years	57.6 ± 9.7	57.4 ± 9.5	57.3 ± 9.6	57.6 ± 9.7	0.61
Women, %	70.7	66.3	58.5	55.7	<0.001
Body mass index, kg/m^2^	24.8 ± 3.7	24.7 ± 3.6	25.0 ± 3.5	25.1 ± 3.5	<0.001
Urban, %	37.3	43.1	67.5	65.8	<0.001
High school or higher education level, %	2.1	3.0	7.3	10.8	<0.001
Farmer, %	46.8	40.3	17.6	14.4	<0.001
Married, %	84.0	86.5	87.7	89.1	<0.001
Income > RMB 35,000, %	8.4	14.0	22.2	26.9	<0.001
Family history of cardiovascular disease, %	18.7	17.3	20.3	22.2	<0.001
History of hypertension, %	21.3	21.6	26.4	26.8	<0.001
Current smoker, %	21.0	22.4	25.7	27.0	<0.001
Current drinker, %	43.7	41.0	46.9	46.3	<0.001
Physical activity, MET·hours/day	15.0 ± 11.2	16.2 ± 11.9	16.4 ± 12.4	16.7 ± 12.4	<0.001
Use of fish oil, %	2.8	3.3	6.5	8.8	<0.001
Use of vitamin supplements, %	2.9	3.8	6.0	9.7	<0.001
Daily tea drinking, %	46.7	39.5	37.0	36.5	<0.001
Daily dairy consumption, %	9.6	10.5	19.2	27.2	<0.001
Daily egg consumption, %	17.1	14.7	19.8	31.3	<0.001
Daily fish consumption, %	3.9	4.6	3.0	5.5	<0.001
Daily fruit consumption, %	10	11.4	23.0	29.3	<0.001
Daily vegetable consumption, %	94.7	91.0	97.5	97.0	<0.001
Daily meat consumption, %	57	60.8	76.8	75.1	<0.001
Daily rice consumption, %	43.1	44.1	45.1	46.1	<0.001

* Values are age-adjusted means ± SD or percentage. MET: metabolic equivalent task.

**Table 2:** Soy consumption and risk of cardiovascular mortality in Chinese adults with type 2 diabetes.

	Never	Monthly	1–3 days/week	≥4 days/week	*p* Trend
	0 g/day *	3.6 g/day	11.5 g/day	15.3 g/day	
Total CVD					
Person-year	18,206	49,541	107,870	26,693	
No. of cases	216	495	754	161	
Model 1	1.00	0.87 (0.74, 1.02)	0.60 (0.52, 0.70)	0.49 (0.40, 0.60)	<0.001
Model 2	1.00	0.92 (0.78, 1.09)	0.89 (0.75, 1.05)	0.77 (0.62, 0.96)	0.02
CHD					
No. of cases	79	158	302	56	
Model 1	1.00	0.76 (0.58, 0.99)	0.66 (0.52, 0.85)	0.47 (0.33, 0.66)	<0.001
Model 2	1.00	0.78 (0.59, 1.03)	0.81 (0.62, 1.07)	0.56 (0.39, 0.81)	0.007
Acute myocardial infarction					
No. of cases	53	97	132	23	
Model 1	1.00	0.69 (0.49, 0.96)	0.43 (0.31, 0.59)	0.29 (0.18, 0.47)	<0.001
Model 2	1.00	0.74 (0.52, 1.06)	0.68 (0.47, 0.98)	0.44 (0.26, 0.73)	0.004
Stroke					
No. of cases	98	232	339	74	
Model 1	1.00	0.89 (0.7, 1.13)	0.59 (0.47, 0.74)	0.50 (0.37, 0.67)	<0.001
Model 2	1.00	0.97 (0.76, 1.25)	0.99 (0.77, 1.27)	0.92 (0.66, 1.27)	0.63
Hemorrhagic stroke					
No. of cases	51	143	159	30	
Model 1	1.00	1.05 (0.76, 1.44)	0.53 (0.39, 0.73)	0.39 (0.25, 0.62)	<0.001
Model 2	1.00	1.04 (0.75, 1.46)	1.03 (0.73, 1.46)	0.82 (0.51, 1.33)	0.33
Ischemic stroke					
No. of cases	29	59	113	26	
Model 1	1.00	0.77 (0.5, 1.21)	0.67 (0.45, 1.01)	0.58 (0.34, 0.99)	0.07
Model 2	1.00	0.95 (0.6, 1.52)	0.98 (0.62, 1.55)	0.90 (0.51, 1.60)	0.76

* Corresponding to soybean equivalents [12]. Abbreviations: CVD, cardiovascular disease; CHD, coronary heart disease. Model 1: HRs (95% CIs) were adjusted for age. Model 2: HRs (95% CIs) were further adjusted for sex, study area, body mass index, family history of CVD, history of hypertension, drinking status, smoking status, education level, household income, occupation, marital status, physical activity, vitamin supplement use, fish oil use, consumption of dairy foods, tea, eggs, fresh fish, fresh fruits, fresh vegetables, meat, and rice.

**Table 3:** Multivariable-adjusted risks for the associations between soy product consumption and cardiovascular mortality by age, sex, body mass index (BMI), smoking status, drinking status, region, and hypertension status among people with type 2 diabetes at baseline.

	Cases	HR (95% CI) *	*p* Value for Interaction
Age<60			
Yes	15,662	0.79 (0.52, 1.20)	0.50
No	10,477	0.76 (0.59, 0.98)	
Men			
Yes	10,133	0.69 (0.49, 0.97)	0.86
No	16,006	0.85 (0.63, 1.14)	
BMI < 25			
Yes	13,573	0.57 (0.43, 0.76)	0.03
No	12,566	1.12 (0.80, 1.56)	
BMI < 23			
Yes	7663	0.63 (0.44, 0.90)	0.12
No	18,476	0.89 (0.68, 1.18)	
Current smoking			
Yes	6435	0.78 (0.61, 1.00)	0.94
No	19,704	0.74 (0.48, 1.14)	
Current drinking			
Yes	11,778	0.81 (0.61, 1.07)	0.86
No	14,361	0.70 (0.49, 0.99)	
Urban			
Yes	15,267	0.83 (0.59, 1.16)	0.11
No	10,872	0.67 (0.49, 0.92)	
Hypertension			
Yes	6481	0.91 (0.62, 1.33)	0.33
No	19,658	0.73 (0.56, 0.95)	

* Hazard ratios (HRs) and 95% confidence intervals (CIs) were for the extreme comparison (≥4 days/week vs. never) and adjusted for age, sex, study area, body mass index, family history of cardiovascular disease, history of hypertension, drinking status, smoking status, education level, household income, occupation, marital status, physical activity, vitamin supplement use, fish oil use, consumption of dairy foods, tea, eggs, fresh fish, fresh fruits, fresh vegetables, meat, and rice.

## Data Availability

Data were obtained from the CKB Data Access System (www.ckbiobank.org (accessed on 20 June 2020), DAR-2019-00016).

## Supplementary Materials

The following are available online at https://www.mdpi.com/article/10.3390/nu13082513/s1, Table S1: Sensitivity analyses of soy consumption and risk of cardiovascular mortality in Chinese adults with type 2 diabetes.

## Abbreviations

CDC, Chinese Center for Disease Control and Prevention; CKB, Chinese Kadoorie Biobank; FFQ, food frequency questionnaire.
