# Supplementary material for: Dietary Soy Consumption and Cardiovascular Mortality among Chinese People with Type 2 Diabetes
Source: Nutrients. 2021 Jul 23;13(8):2513. doi: 10.3390/nu13082513 (PMC8398979; doi:10.3390/nu13082513)
Supplement: Supplementary file 1 [file nutrients-13-02513-s001.zip › nutrients-1291564-supplementary.pdf]

Supplementary Table S1. Sensitivity analyses of soy consumption and risk of cardiovascular mortality in Chinese adults with type 2 diabetes.

	Never<br>0	≥ 4 days/wk<br>15.3 g/day
Total CVD		
Model 2*	1.00	0.77 (0.62, 0.96)
Model 2 + Further adjusted for years of diabetes history	1.00	0.77 (0.59, 1.01)
Model 2 + Excluding participants who died within the first 3 years of follow-up	1.00	0.78 (0.60, 0.99)
Model 2 + Further adjusted for antidiabetic and lipid-lowering therapy	1.00	0.78 (0.63, 0.97)

*Model 2: HRs (95%CIs) were adjusted for sex, study area, body mass index, family history of CVD, history of hypertension, drinking status, smoking status, education level, household income, occupation, marital status, physical activity, vitamin supplement use, fish oil use, consumption of dairy foods, tea, eggs, fresh fish, fresh fruits, fresh vegetables, meat, and rice.
